# Gastrointestinal Behçet-like syndrome as an initial manifestation in myelodysplastic syndrome with double trisomy of chromosomes 8 and 20 and a 20q deletion

**DOI:** 10.1093/rap/rkag044

**Published:** 2026-04-03

**Authors:** Megumi Murakami, Kentaro Kuzuya, Masahiro Misago, Yoshinobu Matsuura, Yoshiki Murakami, Dai Aoki, Shinichiro Nakao, Mototoshi Ito, Norihiko Yamaguchi, Hiroyuki Yamane

**Affiliations:** Department of Emergency and General Medicine, Ikeda City Hospital, Ikeda, Osaka, Japan; Department of Rheumatology, Saiseikai Senri Hospital, Suita, Osaka, Japan; Department of Emergency and General Medicine, Ikeda City Hospital, Ikeda, Osaka, Japan; Department of Rheumatology, Saiseikai Senri Hospital, Suita, Osaka, Japan; Department of Respirology, Saiseikai Senri Hospital, Suita, Osaka, Japan; Department of Emergency and General Medicine, Ikeda City Hospital, Ikeda, Osaka, Japan; Department of Emergency and General Medicine, Ikeda City Hospital, Ikeda, Osaka, Japan; Department of Emergency and General Medicine, Ikeda City Hospital, Ikeda, Osaka, Japan; Department of Respirology, Saiseikai Senri Hospital, Suita, Osaka, Japan; Department of Respirology, Saiseikai Senri Hospital, Suita, Osaka, Japan

Key message

- Gastrointestinal Behçet-like syndrome in MDS with double trisomy 8 and 20 and a 20q deletion.

Dear Editor, Behçet’s syndrome is a chronic inflammatory disease with a relapsing and remitting course and multiple organ involvement. Among these manifestations, gastrointestinal involvement is characterized by deep ulcers around the ileocecal valve and can be life-threatening [1]. Recent reports have shown that gastrointestinal Behçet-like syndrome overlaps with myelodysplastic syndrome (MDS), a haematologic malignancy characterized by cytopenias and chromosomal abnormalities. Notably, trisomy 8 is present in >80% of MDS with Behçet-like syndrome [2] and these patients often present with gastrointestinal and haematologic involvement [3]. Although this condition has been increasingly recognized, cases of additional chromosomal abnormalities and their effects on clinical presentation remain unclear. Herein we report a case of gastrointestinal Behçet-like syndrome as the initial manifestation of MDS with complex chromosomal abnormalities, double trisomy of chromosomes 8 and 20 and a 20q deletion.

A 77-year-old man presented to our hospital with a sudden onset of general fatigue and high fever (39.0°C). On physical examination, he had no abdominal pain and no suspicious findings of uveitis, oral aphthous, genital ulcers or arthritis. Rapid antigen tests for COVID-19 and influenza were negative. The blood exam revealed leucocytosis (white blood cell count 17 290/μl, neutrophils 79.4%), mild anaemia (haemoglobin 10.6 g/dl, mean corpuscular volume 95.1 fl), normal platelet count (17.3 × 106/μl) and elevated CRP (10.5 mg/dl), with no blasts or other remarkable abnormality. He was admitted to our hospital for further assessment. Blood culture, QuantiFERON-TB test and serum autoantibodies (ANA and ANCA) were negative. Contrast-enhanced CT showed wall thickening of the terminal ileum. The second day after admission he developed haematochezia. Gastrointestinal endoscopy on the next day revealed small duodenal ulcers and multiple well-defined ulcers with some deep depressions extending from the ileocecal valve to the terminal ileum (Fig. 1A). Assessment of the biopsied tissue of the intestinal ulcers showed negative results in acid-fast bacilli culture and CMV-infected cells. Histopathological findings revealed no malignancy, but there were infiltrations of neutrophils around small vessels and infiltration of neutrophils and lymphocytes from the lamina propria to the submucosal layer. Although typical findings of Behçet’s syndrome were absent, the widespread distribution of gastrointestinal ulcers extending beyond the ileocecal valve has been reported as a characteristic of gastrointestinal Behçet-like syndrome in MDS with trisomy 8 [4], which implied this differential diagnosis. As his general condition deteriorated with severe inflammation (maximum CRP 38 mg/dl) and paralytic ileus, treatments with intravenous methylprednisolone (125 mg/day) followed by high-dose corticosteroids (prednisolone 1 mg/kg/day) and infliximab (IFX, 5 mg/kg) were introduced, which resulted in disease improvement. A follow-up colonoscopy 30 days after admission showed improvement of the ulcers. He was discharged 45 days after admission. However, his disease relapsed at 3 months after admission with right lower abdominal pain and elevated CRP under treatment with IFX, tacrolimus and medium-dose prednisolone. Therefore, upadacitinib was introduced, after which his symptoms ameliorated. Also, his pancytopenia stabilized for 4 months thereafter (Fig. 1C).

**Figure 1:**
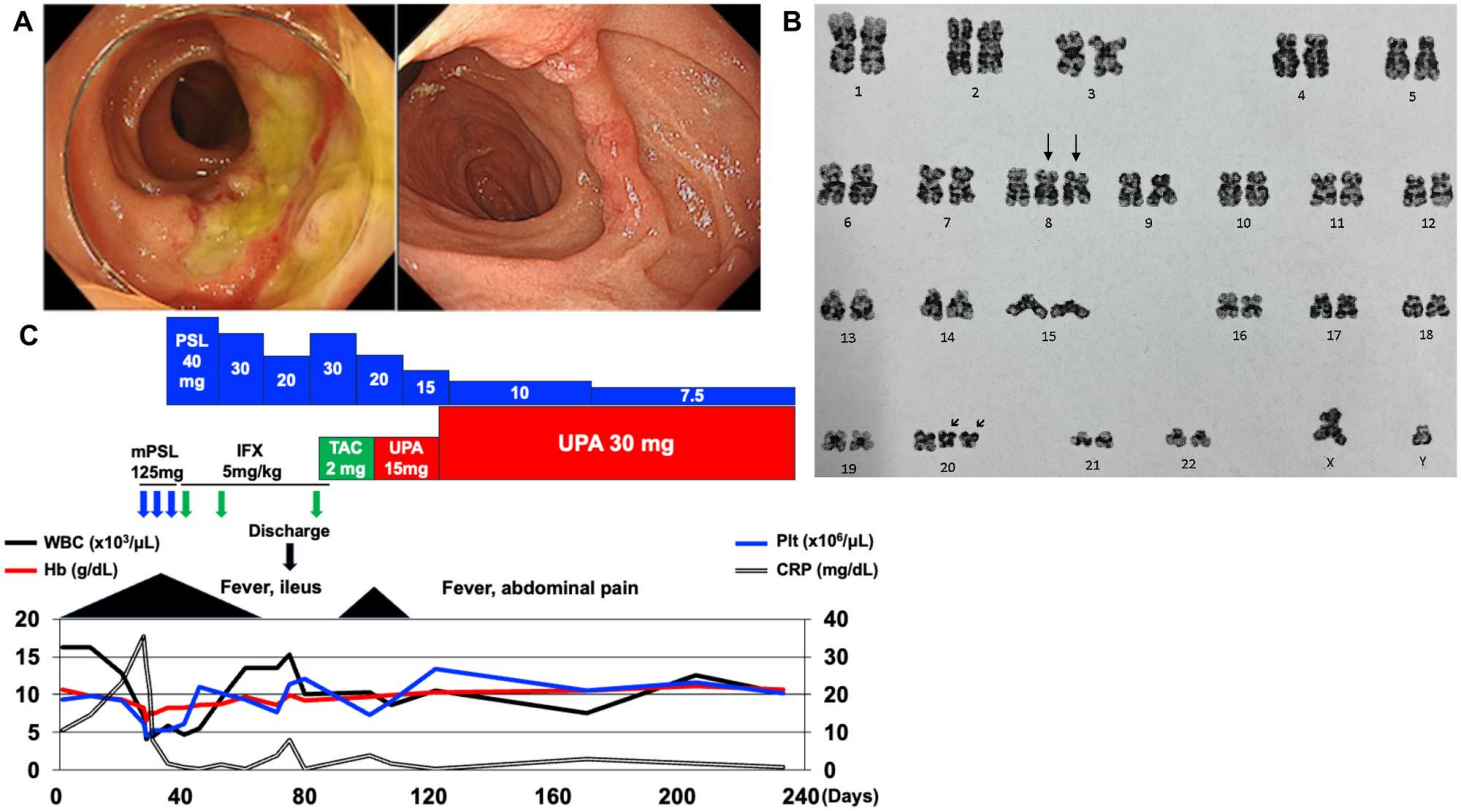
Patient’s clinical data. **(A)** Representative gastrointestinal endoscopic findings. Left: deep ulcers in the terminal ileum. Right: small duodenal ulcers. **(B)** G-banding analysis of bone marrow cells. The patient showed an abnormal karyotype: 48, XY, +8, +20, del(20)(q11.2q13.3)×2. Arrows indicate trisomy 8 and trisomy 20 with a 20q deletion, respectively. **(C)** Patient’s clinical course. Days: days after admission; WBC: white blood cell count; Hb: haemoglobin; Plt: platelet count; mPSL: methylprednisolone; PSL: prednisolone; TAC: tacrolimus; UPA: upadacitinib

As he developed pancytopenia after hospitalization, a bone marrow examination was performed 2 months after admission. Myelogram findings were as follows: normocellular, myeloblasts 2% and dysplastic findings with ringed sideroblasts 18%, with no dysplasia in granuloblasts or megakaryocytes. G-banding of bone marrow cells showed an abnormal karyotype: 48, XY, +8, +20, del(20)(q11.2q13.3)×2 (Fig. 1B). He was diagnosed with MDS with ring sideroblasts and single lineage dysplasia (MDS-RS-SLD).

To our knowledge, this is the first report of gastrointestinal Behçet’s-like syndrome in MDS with double trisomy 8 and 20 and a 20q deletion. Recently, cases of gastrointestinal Behçet’s-like syndrome with double trisomy 8 and 9 have been reported [5]. Compared with our case, the reported case had mucosal and haematological involvement before the diagnosis. In contrast, both cases were treatment-resistant but responded well to Janus kinase (JAK) inhibitor.

In MDS, a worse survival rate (17 months, median) was reported in trisomy 8 with more than two other chromosomal abnormalities, such as in our case, than in isolated trisomy 8 (22 months, median) [6]. Therefore, it is necessary to introduce effective treatments for both inflammatory and haematological aspects in this critical setting. In that regard, JAK inhibitors have been shown to have favourable efficacy on refractory Behçet’s syndrome [5]. Also, a selective JAK2 inhibitor, ruxolitinib, modulates epigenetic regulation of myeloid differentiation [7] and has shown improvement of survival in MDS when combined with azacitidine [8], suggesting the potential therapeutic value of JAK inhibitors in this complex disease state.

In conclusion, we report a case of gastrointestinal Behçet-like syndrome in MDS with double trisomy of chromosomes 8 and 20 and a 20q deletion. It is important to consider Behçet-like syndrome with MDS as a differential diagnosis of gastrointestinal ulcers of unknown aetiology, even without typical manifestations such as oral aphthous. Accumulation of cases is warranted to delineate the disease characteristics of Behçet-like syndrome with MDS that has chromosomal abnormalities beyond trisomy 8, such as our case.

## Data Availability

All relevant data are included in the article.
